# Ecological niche modeling to determine potential niche of Vaccinia virus: a case only study

**DOI:** 10.1186/s12942-017-0100-1

**Published:** 2017-08-07

**Authors:** Claire A. Quiner, Yoshinori Nakazawa

**Affiliations:** Poxvirus and Rabies Branch, Division of High-Consequence Pathogens and Pathology (DHCPP), National Center for Emerging and Zoonotic Infectious Diseases (NCEZID), US Centers for Disease Control and Prevention, Atlanta, GA USA

**Keywords:** Vaccinia, Emerging infectious diseases, Ecological niche model, Orthopoxvirus, Case-only study

## Abstract

**Background:**

Emerging and understudied pathogens often lack information that most commonly used analytical tools require, such as negative controls or baseline data; thus, new analytical strategies are needed to analyze transmission patterns and drivers of disease emergence. Zoonotic infections with Vaccinia virus (VACV) were first reported in Brazil in 1999, VACV is an emerging zoonotic *Orthopoxvirus*, which primarily infects dairy cattle and farmers in close contact with infected cows. Prospective studies of emerging pathogens could provide critical data that would inform public health planning and response to outbreaks. By using the location of 87-recorded outbreaks and publicly available bioclimatic data, we demonstrate one such approach. Using an ecological niche model (ENM) algorithm, we identify the environmental conditions under which VACV outbreaks have occurred, and determine additional locations in two affected countries that may be susceptible to transmission. Further, we show how suitability for the virus responds to different levels of various environmental factors and highlight the most important factors in determining its transmission.

**Methods:**

A literature review was performed and the geospatial coordinates of 87 molecularly confirmed VACV outbreaks in Brazil were identified. An ENM was generated using MaxENT software by combining principal component analysis results of 19 bioclim spatial layers, and 25 randomly selected subsets of the original list of 87 outbreaks.

**Results:**

The final ENM predicted all areas where Brazilian outbreaks occurred, one out of five of the Colombian outbreak regions and identified new regions within Brazil that are suitable for transmission based on bioclimatic factors. Further, the most important factors in determining transmission suitability are precipitation of the wettest quarter, annual precipitation, mean temperature of the coldest quarter and mean diurnal range.

**Conclusion:**

The analyses here provide a means by which to study patterns of an emerging infectious disease and identify regions that are potentially suitable for its transmission, in spite of the paucity of high-quality critical data. Policy and methods for the control of infectious diseases often use a reactionary model, addressing diseases only after significant impact on human health has ensued. The methodology used in the present work allows the identification of areas where disease is likely to appear, which could be used for directed intervention.

**Electronic supplementary material:**

The online version of this article (doi:10.1186/s12942-017-0100-1) contains supplementary material, which is available to authorized users.

## Background

Zoonotic pathogens, including Ebola virus, H1N1, MERS and SARS [1–5], impose significant threats to human health and are projected to increase in their distribution and impact in coming years [5]. Currently, 61% of all pathogens that infect humans are zoonotic and 75% of emerging disease pathogens are zoonotic in origin [6]. This pattern is driven in part by novel interactions between humans and previously undisturbed environments, and can be attributed to human modifications, land-cover change, climate change, unplanned urbanization and human migration [5].

*Vaccinia virus* (VACV) is one such example of an emerging, zoonotic pathogen. VACV is an *Orthopoxvirus* and is closely related to the virus that causes smallpox (*Variola virus*). VACV was used as the vaccine against smallpox during eradication efforts, but more recently, human infections of zoonotic origin have been reported [7–9] in Brazil, India [7] and Mongolia [10]. The natural history of VACV and its transmission cycle is not known, but several wild and peri-domestic species of mammals have shown evidence of orthopoxvirus infection, including horses, coatis, opossums, monkeys and rodents, which could be involved in the maintenance of the virus in nature [11–16]. In South America, the first VACV outbreak of zoonotic origin was identified in Brazil in 1999 [17] and all documented VACV outbreaks on the continent since that year have been associated with dairy farms in Brazil [18–21] or Colombia [22]. During an outbreak, the virus is presumably spread throughout a farm by direct cow-to-cow contact or via milkers who develop lesions on their hands and spread the virus to others during milking. The virus could be transmitted to neighboring farms by sharing infected cattle for breeding practices and/or infected milkers. Secondary human cases of VACV without direct physical contact with infected cattle, have also been reported [17, 23]. VACV is not a mandatory reportable disease and the current surveillance system is not designed to capture these infections. Further, only a limited number of epidemiologic studies have been conducted, which restricts the ability to estimate the burden of the disease and the use of other analytical approaches to research transmission patterns and risk factors that would aid in its control.

VACV infection causes moderate to severe illness in humans and reduces milk production in cows; disease manifestation in humans includes pruritus at the site of infection, papules, vesicles, and pustules surrounded by erythema and induration as well as fever, headache, exhaustion, enlarged lymph nodes, and malaise; symptoms last for up to 30 days [21]. Experimentally infected cows show symptoms that last 1–32 days post inoculation (dpi), whereby vesicles, papules and ulcers form on teats, and in some cases the muzzle as well, and eventually scar. Milk production is affected by infection as mastitis begins early in infection and remains through the entirety of the disease. Milk volume drops by more than 70% by 3 dpi and milk quality, measured by somatic cell count (SSC), significantly decreases [24]. Studies of milk experimentally contaminated with VACV showed a major reduction of infective viral particles (>94%), after the pasteurization process but a few were still infective [25].

The dairy industry in Brazil is currently the world’s 5th largest milk producer and is rapidly increasing. There are over 1 million dairy cattle farms in Brazil, which are heavily concentrated in the states which have experienced VACV outbreaks (Minas Gerais, São Paulo, Goiás, and Rio Grande do Sul [26]). Studies of milk experimentally contaminated with VACV showed a major, but not complete, reduction of infective viral particles (>94%) after the pasteurization process [25], this opens the possibility for viral spread through consumption of milk.

Public health control of emerging pathogens is challenging when the origin and basic risk factors for pathogen acquisition are not well understood. The mechanism by which VACV is maintained in nature, cows become infected, transmission patterns, attack rate and basic risk factors are still unknown. In lieu of opportunities to collect more data from larger outbreaks or formal epidemiological studies, this work attempts to utilize the existing and publicly available information to gain insight into this emerging threat. Based on the premise that pathogen circulation depends, in part, on certain environmental conditions, identifying and mapping those conditions can be used to hypothesize the distribution of a pathogen across the landscape [27]. Here, we aim to identify at-risk regions for VACV transmission in Brazil and Colombia by determining the environmental factors common among locations in which outbreaks have been recorded, and to identify the most relevant bioclimatic factors affecting its transmission.

## Methods

### Input data

#### Outbreak occurrence data

A literature search was performed to create a list of VACV outbreaks and their geographical coordinates. The search was conducted in PubMed, was restricted to articles in English and used the following search terms: Bovine Vaccinia, Vaccinia virus, Bovine Associated Vaccinia, or Brazilian Vaccinia. References within articles identified by this search were reviewed for other publications that were not found in the original. Results were further supplemented with publications suggested by subject matter experts including Brazilian researchers familiar with local publications. Inclusion criteria for an outbreak were (1) the outbreak occurred in Brazil, (2) the etiologic agent was confirmed as VACV via molecular diagnostics, and (3) the article noted the municipality in which the outbreak occurred. The centroid of each municipality was then used to represent the location of disease occurrence. The complete list of outbreaks used for modeling is listed in Additional file 1 and summarized by state in Table 1.

**Table 1:** Brazilian outbreaks of VACV by state

State	# of VACV outbreaks
Bahia	1
Espírito Santo	9
Goiás	3
Maranhão	1
Mato Grosso	2
Minas Gerais	33
Rio de Janeiro	22
Rio Grande do Sul	1
São Paulo	15

Number of recorded VACV outbreaks in each Brazilian state

Information concerning reported cases of VACV in Colombia is more limited: they have occurred in the municipalities of Medina, Puerto Salgar (INS Personal Communications) and Valapara**í**so [22]. Additionally, cow samples from the departments of Casanare and Santander have been found to be positive (INS Personal Communications).

#### Climatic data

At broad scales, climatic variables have been used in ecological niche models to find non-random associations between occurrences and environmental conditions at those locations to estimate distribution of many infectious diseases [28, 29]. Here, we used climatic datasets from WorldClim, http://www.worldclim.org/bioclim, which provide fine-scale data of various environmental factors for the entire world, including minimum, maximum, and average temperature, annual precipitation, as well as seasonal estimates for each factor. These datasets are publicly available through 19 bioclimatic spatial layers [30] and are offered in four resolutions. A visual comparison of each resolution’s pixel size to the average municipality size was performed to select the most adequate spatial resolution to fit the precision of the VACV occurrence data. To reduce dimensionality and auto-correlation between variables, Principal Components (PC) were calculated based on the 19 bioclim layers in ArcMap, v. 10.3.1 over the total area of interest (Colombia and Brazil) [31] (Table 2).

**Table 2:** PCA results

Bio clim layer	PC 1	PC 2	PC 3	PC 4	PC 5
PWQ	*0.6356*	*0.5965*	*0.2536*	0.0680	−0.2815
MTCQ	*0.2760*	*0.2158*	−0.8614	−0.1089	*0.3413*
AP	*0.2166*	0.0951	*0.0801*	*0.6262*	*0.2729*
TS	0.0830	0.1829	−0.0215	−0.5473	−0.3041
PS	0.0750	0.0312	0.0203	*0.2270*	0.1051
MTWaM	0.0360	0.1909	*0.4181*	−0.4064	*0.7729*
ISO	0.0306	−0.0205	−0.0466	−0.0061	−0.0659
PDQ	0.0255	−0.0257	−0.0134	0.0306	−0.0361
PDM	0.0238	0.0547	−0.0042	−0.1760	−0.0939
MTCM	0.0236	−0.0199	−0.0302	0.0310	−0.0683
PCQ	0.0161	−0.0165	−0.0120	0.0315	−0.0308
TAR	0.0101	−0.0126	−0.0006	0.0275	−0.0036
PWaQ	0.0087	−0.0078	−0.0159	0.0340	−0.0390
MTWaQ	0.0071	−0.0047	−0.0045	−0.0161	0.0057
MTDQ	0.0066	−0.0116	−0.0029	0.0671	−0.0441
PWM	−0.0048	−0.0251	0.0070	0.0725	0.0518
AMT	−0.0069	−0.0040	0.0270	0.0338	0.0292
MTWQ	−0.0240	0.0089	0.0437	0.0732	0.0217
MDR	−0.6747	*0.7154*	−0.0722	*0.1510*	−0.0356
% of eigen values	66.9938	92.3237	97.2055	98.8945	99.6106

Eigen vectors and values

Listed are the Eigen vectors, indicating the contributions of each bioclim layer to the 5 principle component (PC) layers, used in MaxENT modeling. The three largest contributors to each layer are highlighted in italics. Eigen values listed in the last row indicate the amount of heterogeneity that each PC layer accounts for *PWQ* precipitation of wettest quarter, *MTCQ* Mean Temperature of Coldest Quarter, *AP* annual precipitation, *TS* Temperature Seasonality (standard deviation * 100), *PS* Precipitation Seasonality (Coefficient of Variation), *MTWaM* maximum temperature of the warmest month, *ISO* isothermability (Bio2/Bio7) * (100), *PDQ* Precipitation of Driest Quarter, *PDM* precipitation of the driest month, *MTCM* minimum temperature of the coldest month, *PCQ* Precipitation of Coldest Quarter, *TAR* Temperature Annual Range (MTWaM–MTCM), *PWaQ* Precipitation of Warmest Quarter, *MTWaQ* Mean Temperature of Warmest Quarter, *MTDQ* Mean Temperature of Driest Quarter, *PWM* precipitation of the wettest month, *AMT* annual mean temperature, *MTWQ* Mean Temperature of Wettest Quarter, *MDR* mean diurnal range

#### Model generation

ENMs have been used to gain understanding of environmental aspects of transmission of diseases and their spatial distribution with limited amounts of available data. Maxent has been shown to be useful in its application to study infectious diseases [32], and to have a higher performance than other similar algorithms [33]. Thus, ENMs were built using MaxENT software [34], which applies the maximum entropy principle, whereby a model is constructed by fitting a probability distribution to the environmental variables, which is closest to uniform and is constrained by parameters associated with the outbreaks; by doing this, MaxENT finds non-random associations between environmental variables and VACV outbreaks via the comparison of environmental conditions at such localities and background conditions within the study area. Here, the default settings in MaxENT (i.e., regularization multiplier = 1.0, 1500 maximum iterations, 10,000 background points, convergence limit = 1025) were used.

In generating an ENM, the choice of a geographic extent in which models will be trained strongly influences the model’s calibration since pseudo-absences can be selected from within this area [35]. An extent that is too large would offer the model too much area where transmission is not possible, resulting in a falsely precise model. A study area too small would not allow for sufficient environmental variability and would limit the selection of pseudo-absences (points where a case has not been reported, but cannot be ruled out) [35]. To address this, six geographic extents were tested—50, 100, 150, 200, 250 and 300 km radius (results for three of them are reported). Given that using a geographic extent that is too big would inflate AUC scores, we tested the extents iteratively, beginning with the smallest, and the one with the highest performance was chosen.

To test the ability of the ENM to predict areas suitable for VACV, the list of outbreaks was divided into two datasets—separated by whether each outbreak fell above or below the median longitude and median latitude of all outbreaks [36]. These datasets, which each contained outbreaks from different quadrants of the study area, were then used as test and training datasets, to test and train model performance.

**Fig. 1:**
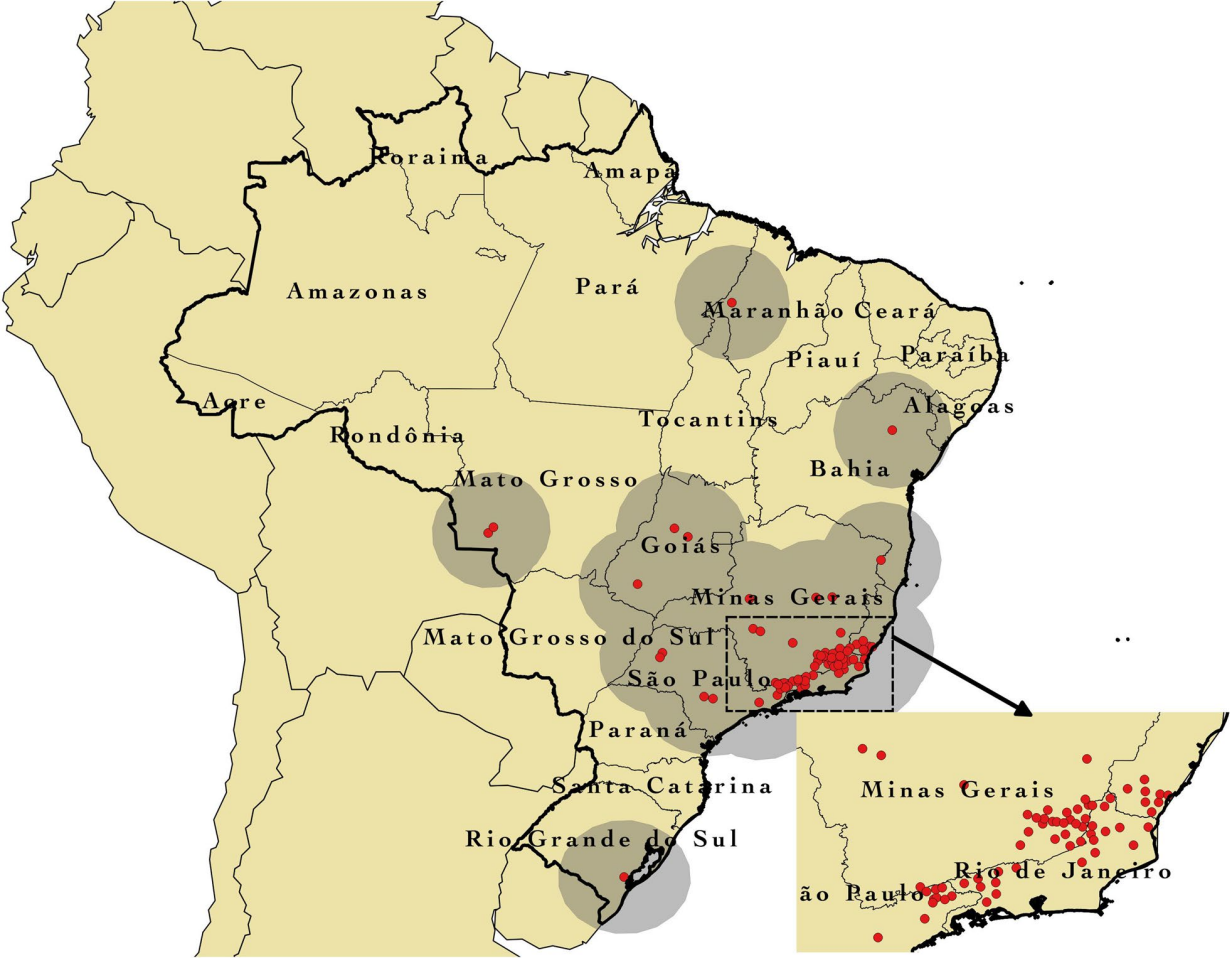
VACV outbreaks in Brazil. *Red points* indicate the centroid of municipalities with confirmed VACV outbreaks. *Grey circles* show the 300 km radius from centroids, which indicates the geographic extent used in MaxENT model. *Inset* most outbreak municipalities were found in southeastern Brazil in the states of Minas Gerais, Espírito Santo, and Rio de Janeiro

Mapping of VACV outbreaks (Fig. 1), identified through the literature search revealed clustering of outbreak reports in southeastern Brazil might be due to reporting bias since surveillance efforts are not uniform across the country. This clustering could interfere with the model performance metrics by means of spatial autocorrelation (i.e., nearby localities have similar environmental conditions and could predict each other) [32]. To address this bias, subsets of the outbreaks were created to generate a more homogeneous spatial representation of the distribution of the disease and correct for spatial autocorrelation. These subsets were created in R Studio, v. 0.99.849 using base packages [37]. To generate a subset, one outbreak was randomly selected as part of the subset and all points within an indicated proximity threshold [either 33 km (0.1°) or 52 km (0.5°)] were removed. Then, a new outbreak was randomly selected from the remaining outbreaks and its neighbors (within the same proximity) were removed. This process was repeated until all outbreaks were assigned to the subset or discarded. This process was repeated 25 times to create 25 subsets with each proximity threshold, either with 33 or 52 km. The resulting subsets contained approximately 70 or 45 outbreaks, respectively. While not every outbreak was included in every subset, each outbreak was included in at least one of the subsets. Another correction for bias was applied by restricting the areas used to train the model to buffered regions around the outbreaks. Pseudo-absences were selected from within these regions, such that the selection bias of the outbreaks was applied to the environmental layers as well [38].

Models were run in MaxENT using each one of the outbreak datasets (33 km subsets, or 52 km subsets) and one set of environmental layers (PC layers clipped to either 50, 100, 150, 200, 250 or 300 km radius). Individual log probability outputs of each model were transformed into binary maps (0 = unsuitable and 1 = suitable using three probability thresholds calculated based on 0, 5, or 10% omission of the training occurrences [39]. Individual binary maps were then combined within each omission level to generate a map that represents model agreement with values ranging from 0 (all models agreed the pixel was unsuitable) to 25 (all models agreed the pixel was suitable) [39]. Finally, the model was projected onto the countries of Brazil and Colombia [40]. This projection was compared to the available geographic information of Colombian VACV outbreaks.

#### Model evaluation and analysis

Models were evaluated using the area under the curve (AUC) of the receiver-operating characteristic (ROC). For medical diagnostics, AUC values 0.5–0.7 are considered low accuracy, values of 0.7–0.9 are accurate and values ≥0.9 are highly accurate [41]. Previous studies selected an AUC of 0.85 as acceptable; given the uncertainty in the precision of the localities (municipalities) we used to generate the model, we would expect higher levels of omission, and therefore considered an AUC above 0.8 as acceptable.

A three-dimensional plot was produced using values from the first three PC layers, to visualize the climatic heterogeneity of the study area and the portions in environmental space occupied by the areas deemed suitable for transmission by the MaxENT model, as compared with the values of the actual outbreaks in Brazil and Colombia.

The PC layers used to make the ENMs contribute differentially to the final model. For each model, MaxENT provides the relative contribution of each variable. The higher the contribution, the more impact a PC layer has on predicting VACV suitability.

These values are derived by default in MaxENT. In brief, the first estimate reflects the increase in regularized gain, which is added to the contribution of the variable. Next, the values of each variable on training presence and background data are randomly permuted. The model is reevaluated on the permuted data, and the resulting drop in training AUC is shown (normalized to percentages). The average contribution of each PC layer across the 25 subsets, and the corresponding standard deviation is reported.

Values for each of the 19 bioclimatic layers were extracted from the areas identified as suitable for transmission in the final MaxENT model. Summary statistics were calculated for each layer. The same statistics are calculated at the points of outbreaks in Colombia and Brazil. These extracted values were also plotted as frequency plots.

Finally, the final model was visually compared to livestock densities as livestock is involved on the virus’ transmission to humans. Estimates of livestock density are provided by the Food and Agriculture Administration of the United Nations (FAO) [42]. The density maps used here are a result of the FAO continuously collecting livestock statistics at sub-national levels. These data are then matched to their administrative boundaries and densities are calculated, accounting for suitable land (i.e., excluding lakes and cities).

## Results

The literature review and selection criteria resulted in the identification of 87 Brazilian municipalities in which VACV outbreaks had occurred, mapped in Fig. 1. Most outbreaks are clustered in southeastern Brazil in the states of Rio de Janeiro, Minas Gerais and São Paulo. Some reports include multiple outbreaks that occurred over a time period [23, 43] while others reported on a single outbreak [44, 45]. Visual comparison of the four spatial resolutions of bioclim layers to the average size of VACV-affected municipalities led to the selection of the 5 arc-min resolution bioclim data for this analysis. At this resolution, pixels in bioclim layers were not considerably smaller or larger than the size of most VACV municipalities.

A principal component analysis of the 19 bioclim layers, revealed that the first five principal components account for 99.6% of the heterogeneity across Brazil and Colombia (Table 2) and were selected for use for in subsequent analyses. Among these five layers, the three largest bioclim contributors for each layer are bolded.

Precipitation of the wettest quarter (PWQ), mean temperature of the coldest quarter (MTCQ) and annual precipitation (AP) were the most important factors for PC 1. For PC 2, in addition to PWQ and MTCQ, mean diurnal range (MDR) was also identified as an important factor.

Multiple ENM models were generated using different combinations of outbreak datasets with environmental layers. These combinations and the resulting AUC values are summarized in Table 3. Only three of the six geographic extents tested are listed here.

**Table 3:** Summary of VACV MaxENT models

MaxENT run	Outbreak dataset	Enviro. layers (radius to centroids)	AUC (SD)	AUC (train)
1	Test v train	PC 1–5 (50 km)	0.64	0.684
2	Train v test	PC 1–5 (50 km)	0.626	0.832
3	Test v train	PC 1–5 (250 km)	0.802	0.935
4	Train v test	PC 1–5 (250 km)	0.848	0.844
5	Subsets, 52 km	PC 1–5 (250 km)	0.803 (0.007)	X
6	Subsets, 33 km	PC 1–5 (250 km)	0.861 (0.003)	X
7	Subsets, 52 km	PC 1–5 (300 km)	0.812 (0.007)	X
8	Subsets, 33 km	PC 1–5 (300 km)	0.867 (0.002)	X
9	Subsets, 52 km	BioClim 1–19 (300 km)	0.873 (0.004)	X
10	Subsets, 33 km	BioClim 1–19 (300 km)	0.907 (0.001)	X
11 (combined datasets)	Subsets, 52 km	PC 1–5 (300 km)	0.95	X

Summary, variables used and resulting AUC values, of MaxENT models run in selecting variables. Subsets had 33 or 52 km, 0.3 or 0.15 decimal degrees in between each outbreak, corresponding to ~52 and 33 km, respectively. MaxENT runs 5–7 were generated using 25 subsets of outbreaks. The AUC values reported here are averages of those AUC’s from those 25 models. Standard deviations are reported in parenthesis

MaxENT models 1–4 were run to determine the ability of an ENM to predict outbreaks by generating the model using a training dataset, which contained approximately half of the total outbreaks, and testing its ability to predict areas suitable for the other half of the outbreaks. Using a geographic extent limited to a 50 km radius surrounding outbreaks yielded models that were not accurate or predictive (Test AUC = 0.64, Train AUC = 0.684). When a buffer of 250 km was used to select the training area, the model improved considerably (Test AUC = 0.802, Train AUC = 0.935). These results indicated that the MaxENT model was capable of predicting outbreak localities by identifying environmental conditions suitable for VACV transmission.

Models were created using each set of the subsets of outbreaks and PC 1–5 layers at a 250 km radius extent. AUC values were higher for those models using subsets generated using 33 km (Model 6 AUC = 0.861) compared with the models, which used 52 km subsets (Model 5 AUC = 0.803). However, this could be due to incomplete elimination of clustering; thus, subsets generated using 52 km were used for modeling, with a slightly larger geographic extent, 300 km (Model 7 AUC = 0.812).

Models 9 and 10 were run using the 19 bioclim variables, which were not reassembled into PC layers. Each of the 19 were clipped to 300 km around each outbreak and models were generated using 52 km (Model 9) and 33 km (Model 10) outbreak subsets. As such, models 7 and 9 are comparable and models 8 and 10 are comparable. In each comparison, the AUC is slightly improved when using 19 bioclim variables, rather than PC layers 1–5. This suggests that use of PC 1–5 removed some information that describes VACV suitability. Five bioclim variables accounted for about 80% of the heterogeneity: isothermability (26.6%), Precipitation of the coldest quarter (15.9%), mean temperature of the driest quarter (15.6%), precipitation seasonality (12.2%), and temperature seasonality (9,7%). The remaining 14 layers each contributed less than 5% each to the heterogeneity of the principle components. One of these variables, precipitation of the coldest quarter, was identified as a key environmental parameter in models, which used PC layers. That the other three variables are different may suggest that without the adjustment provided by principle components, the estimate of these variables is overly emphasized.

**Fig. 2:**
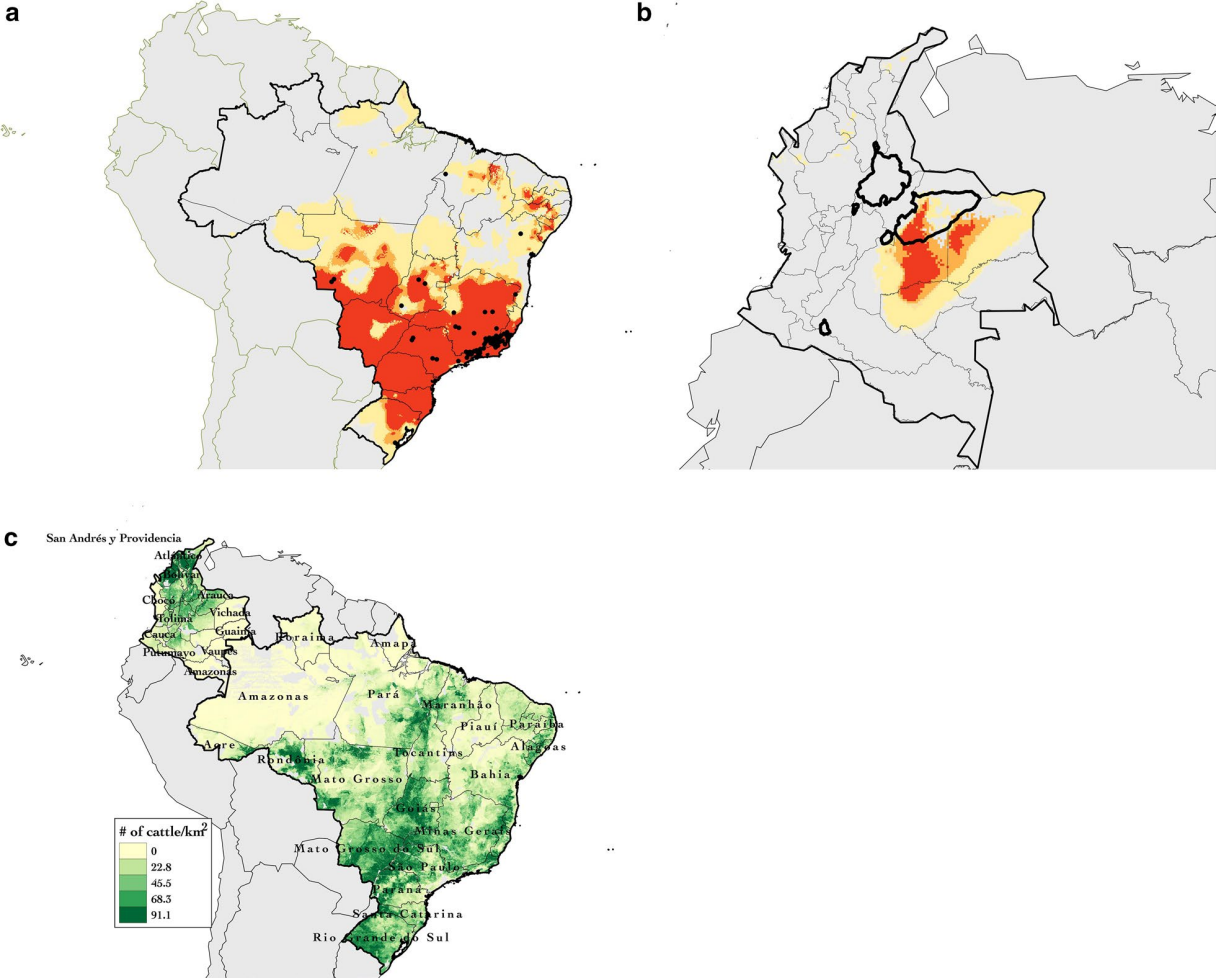
**a** Three omission thresholds—0% = *yellow*, 5% = *orange* and 10% = *red*—of the final MaxENT model projected over Brazil, indicating suitability for VACV transmission. *Black points* show all outbreaks used to generate model, **b** three thresholds of the final MaxENT model projected onto Colombia. The outlines of VACV municipalities (Medina, Valaparaíso and Puerto Salgar) or departments (Casanare and Santander) are outlined in *black,* **c** livestock densities throughout Brazil and Colombia. *Values* represent cattle head densities (values per square kilometer). Country totals are adjusted to FAOSTAT values in 2006

Final models were produced at three thresholds of omission (Fig. 2a). The 0% threshold, in addition to identifying the regions where VACV outbreaks have already occurred, uniquely highlighted new regions of the Mato Grasso, Tocantins, Rio Grande do Sul as well as several central-eastern states, as suitable for transmission. The more conservative 10% threshold identified new states and regions as suitable for transmission, including Santa Catarina, Paraná, and Mato Grosso do Sul. States which have already experienced at least one VACV outbreak, and were identified by the model as suitable to VACV transmission are: Mato Grosso, Rio Grande do Sul, Minas Gerais, Rio de Janeiro, Espírito Santo, Bahia, Goiás, and smaller portions of Maranhão and Bahia. States which have not yet experienced a VACV outbreak, yet were identified as suitable by the model were Mato Grosso do Sul, Paraná, Santa Catarina and smaller portions of Piauí, Ceará, Pernambuco, Alagoas, and Sergipe.

The final models were then projected onto Colombia (Fig. 2b). The known outbreaks that have occurred there are shown by black outline of the most granular geographic extent available: municipalities (Medina, Valaparaíso and Puerto Salgar) or departments (Casanare and Santander). Outbreaks could have occurred anywhere within the outlined regions. The model predicted part of one department, Casanare, and the model, using the 0% threshold, predicted part of one municipality, Medina, as suitable for transmission. The regions in which three of these outbreaks occurred lie outside of the predicted region.

The density of livestock [42] is mapped (Fig. 2c), and many of the Brazilian outbreaks fall within regions of Brazil that have a high density of cattle, i.e., Goiás, Rio de Janeiro, Minas Gerais, and São Paulo. Several areas with high density of livestock are predicted suitable for transmission such as Mato Grosso do Sul, and northwestern Paraná.

**Fig. 3:**
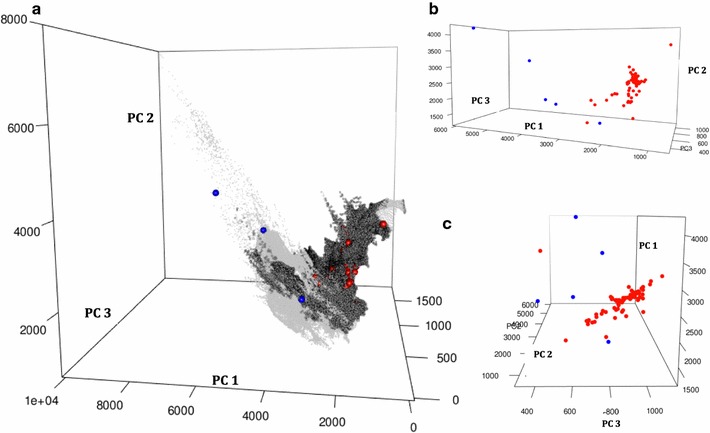
**a**–**c** 3-D plot of values of PC layers 1–3 for Brazil (*red*), Colombia (*blue*), MaxENT predictions (*black*), and background values (*grey*), **b**–**c** different angles of the same plot with only Brazil and Colombia predictions

Figure 3a–c show several angles of the three-dimensional plot of PC 1–3 values. The MaxENT model prediction for suitable ranges for each of these variables is shown in black dots. The values for Brazil (red) fall within this range, as the model accurately predicted the majority of the outbreaks there. Some of Colombia’s outbreaks fall outside of the suitable environments, as predicted by the model. One of these outbreaks is notably discordant for PC 1. Most of Colombia’s outbreaks fell within Brazil’s range for PC layers 2 and 3, but again, varied for PC layer 1. In Fig. 3b, c, the background and MaxENT model conditions were removed for better visualization.

The percent predictive contribution of each PC layer to the final model is listed in Table 4.

**Table 4:** Average contribution of each PC layer to final model

PC layer	Average	SD
PC 1	20.70	2.04
PC 2	53.16	2.82
PC 3	12.71	1.10
PC 4	9.68	1.67
PC 5	3.75	1.10

Average percent contribution of each PC layer, to the final ENM. Average of 25 subsets used to make final model is shown.

**Fig. 4:**
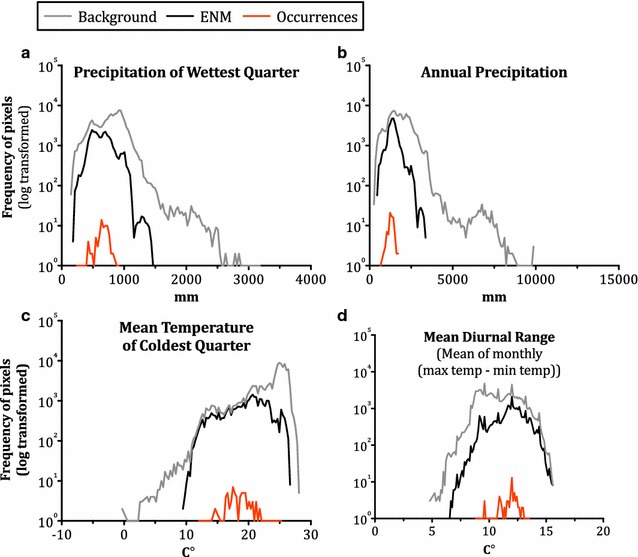
Available values are plotted to demonstrate the key environmental parameters and the ecological niche occupied by VACV, according to MaxENT predictions (*black*), 87 Brazilian outbreaks (*red*), and background (*grey*). On the Y-axis is the number of pixels for each variable and the X-Axis, the bioclim variable indicated

PC layers 1 and 2 collectively contributed over 70% to the final model. The bioclim layers with the highest contribution to PC layers 1 and 2 are precipitation of the wettest quarter (PWQ), mean temp of the coldest quarter (MTCQ), annual precipitation (AP) and mean diurnal range (MDR) and were further analyzed. Plotted in Fig. 4 is the number of pixels deemed as suitable for transmission at each value of the bioclim variable indicated: MaxENT prediction (black line), Brazilian outbreaks (red line). The range for each of these variables, as predicted by MaxENT, is as follows: PWQ (198.12–1546.86 mm), MTCQ (9.5–27.1 °C), AP (467.36–3571.24 mm), and MDR (6.6–15.7 °C). Summary statistics for each of the bioclim layers extracted from each model can be found in Additional file 2 and pixel plots for the remaining 15 bioclim layers are provided in Additional file 3. To summarize the results of the ENM, the environmental factors associated with most of the VACV outbreaks in Brazil include an annual mean temperature of 22.5 °C, mean diurnal temperature range of 11.7 °C, and an annual precipitation of 1493.52 mm.

## Discussion

Ecologic niche modeling was used to identify the actual and potential niche of VACV, an *Orthopoxvirus* which primary affects dairy cattle in Brazil. Subsets of the geographic locations of VACV cases were combined with environmental layers, condensed by a principle component analysis, and were clipped to various extents. The final extent and subset combination was selected by using the model, which produced the highest AUC value. This model was then projected onto a larger geographic range, to include neighboring Colombia, where outbreaks have also occurred. The values of each PC layer as well as the biocim values were extracted at each outbreak location. These values were compared across the two countries. Several methods were used to account for the bias inherent in the outbreak data including: using a 10% threshold of probabilistic estimates, selecting a resolution of environmental data which was not significantly more resolute than the available geographic granularity of the outbreaks, and creating subsets of outbreaks which reduced the effect of clustering.

The result was a model, which identified regions in Brazil where VACV outbreaks have already occurred as well as several new locations within Brazil, which could be vulnerable to a VACV outbreak. Most states identified as suitable for VACV transmission, have regions with a high density of livestock, as this industry is clustered in southern Brazil. Where high density of livestock and suitability for transmission co-localize, the risk of VACV is likely much greater.

The final model predicted a portion of one of the five regions in Colombia, which have confirmed VACV outbreaks. This prediction could be explained in several ways. For example, VACV could have different reservoirs in different regions, i.e., different mammal species maintain the virus in nature, each with different ecologic/environmental requirements. An argument for multiple reservoirs is supported given the variation in small rodent species found to have evidence of infection with orthopoxviruses in Brazil [46]. Additionally, poxviruses can infect several species of animals i.e., *Monkeypox virus* in pouched rats, [47], prairie dogs, squirrels [48], and dormice [49], among others; *Cowpox virus* in voles [50], llamas [51], mice [52], cats [53] among other species of animals. Consistent with this is the results of the 3D graph, which allows for comparison of the ecologic niche that VACV occupies across the two countries. The discordance for PC 1 suggests that VACV occupies a different climate space in Colombia, as compared with Brazil. Further, suitable environmental conditions alone are insufficient for transmission of VACV, as the suitable area would also have to be occupied by its reservoir, and pathogen distribution is also restricted by geographic barriers, mobility, and human intervention [54]. Finally, the limited number of outbreaks that have been recorded in Colombia leaves the predictive capability of the Brazil model for Colombia, inconclusive. Only a few VACV infections in Colombia have been reported since 2014; all of which have had contact with cows. It is possible that cases are happening in areas of high livestock density in Colombia, but they go unreported since the surveillance system is not designed to capture them. Given the differences in the available data from Brazil and Colombia, (i.e., both geographically biased, but Brazil having many more years detecting and reporting) conclusions about the different niches for VACV in these places, are limited.

Key bioclimatic indicators for this disease have also been identified by the model: PWQ, MTCQ, AP and MDR.

Several limitations exist for this type of modeling. To generate the ENM reported here, the centroids of municipalities, which have experienced a VACV outbreak, not the actual farm, were used. Significant environmental heterogeneity across the municipality would reduce the precision of the final model. Further, the outbreaks used to make the model are a result of a literature review, which do not represent reports from active surveillance of disease in humans or cattle; therefore, there is an inherent bias that could over-represent the geographic areas that routinely report and test for VACV. Several measures have been taken to minimize the potential effects of these biases, including the use of subsets, limiting geographic extents, and the use of a 10% threshold to make conservative estimates. Improvements in either the specificity of coordinates used and in data collection methods would likely improve model prediction. Implementing a surveillance program for VACV would improve the precision and number of cases and outbreaks. This would, in turn, improve model accuracy and predictive capability. Additionally, overcoming barriers for reporting cases (i.e., fear of closing farms) would aid in surveillance efforts. High-quality occurrence data would also allow the use of relevant non-climatic factors such as land use, trade data, milker travel records, and other sources of environmental data (i.e., satellite imagery) at higher spatial and temporal resolutions to refine the models and broaden our understanding of the ecology of this pathogen.

Despite their limitations, the data presented here could provide valuable information to public health officials in protecting human health proactively; areas where the ecological niche predicts suitable environments for transmission could be targeted by education campaigns to inform local farmers of symptoms and warn against sharing of cows with these farms, early symptoms in cows, horses, and humans, and encourage methods to prevent its spread such as improved sanitation and ill cattle isolation. Similarly, future epidemiological and ecological studies could focus on these areas and study the local species and their potential role in the maintenance of VACV in nature. Given the prediction capabilities of the model in Brazil, in its current state, this model would be of most use in Brazil, for these purposes.

Public engagement and a participatory process, inclusive of all stakeholders: farmers, milk consumers, planning officials, public health personnel, and community organizations, would improve the quality and impact of all interventions aimed at preventing and mitigating harm from outbreaks.

This information is increasingly relevant in context of the growing dairy industry in Brazil. An estimated 30% of the total milk production in Brazil in 2014, 36 billion liters, was under informal methods, or not under the inspection of government officials [26]. Moreover, populations in VACV affected regions of Brazil practice a traditional cheese making process which uses unpasteurized milk [55], whereby virus in milk may not be entirely inactivated. There is a documented case of a human patient, without any direct contact with cows, who developed VACV lesions of the mouth [56] suggesting a potential risk for transmission via consumption of infected milk.

Most VACV publications, to date, are descriptions of outbreaks or reported cases [43, 57–61], and a few others describe controlled experiments using VACV to infect cows or mice [62]; however, to the best of our knowledge, there are no publications describing the suitable environmental conditions for the transmission of VACV. The recent VACV reports from Colombia highlights the potential for VACV, or other poxviruses, to cause human and animal disease in other countries. Further, current events have illuminated the threat of spread of infectious diseases, which in past decades may have been isolated to a certain region, but now have potential to spread globally in a relatively short period of time [63, 64]. Finally, herd immunity to poxviruses is dissipating due to smallpox vaccination no longer being routine.

## Conclusions

The study of emerging diseases presents a unique set of challenges, several of which are highlighted in work presented here: selection bias, specificity of data and limited information, are among them. In addition, lack of basic information about a disease, such as a complete host range and transmission patterns, leaves prevention efforts with little direction. Finally, the lack of a surveillance, cohort or case control study, limits the analytical methods that could be used. Here, we sought to address these challenges by applying an ecological niche model as a proof of concept to demonstrate ways to spatially predict VACV outbreaks. The analyses here provide a means by which to study the patterns of an emerging infectious disease, and regions that are potentially at risk for it, in spite of the paucity of critical data and limitations described above.

Policy and methods for the control of infectious diseases often use a reactionary model, addressing diseases only after significant impact on human health has ensued. Here, we provide a means to predict where the disease is likely to appear, providing a map for effective prevention. Contemporary events [64, 65] strongly indicate the need for the study of emerging and neglected diseases despite the implicit hurdles. Global developments over the last century have given many infectious diseases a new landscape and offer them boundless immune susceptible organisms. In the pursuit to counteract these measures, current strategies will not suffice in protecting human health. We must look for novel solutions and means to prevent and mitigate infectious disease epidemics.

## Additional files

**Additional file 1.** List of Brazilian outbreaks used to generate ENM, with references.

**Additional file 2.** Summary statistics of key bioclim values as predicted by MaxENT modeling, using a 10% threshold, and what is indicated at the points of VCAV occurrence in Brazil and Colombia. Temperatures are reported in degrees Fahrenheit and precipitation in mm. AMT, annual mean temperature; MDR, mean diurnal range; ISO, isothermability (Bio2/Bio7)*(100); TS, Temperature Seasonality (standard deviation *100); MTWaM; maximum temperature of the warmest month; MTCM, minimum temperature of the coldest month; TAR, Temperature Annual Range (Bio5-Bio6); MTWQ, Mean Temperature of Wettest Quarter; MTDQ, Mean Temperature of Driest Quarter; MTWaQ, Mean Temperature of Warmest Quarter; MTCQ, Mean Temperature of Coldest Quarter; AP, annual precipitation; PWM, precipitation of the wettest month; PDM, precipitation of the driest month; PS, Precipitation Seasonality (Coefficient of Variation); PWQ, Precipitation of Wettest Quarter; PDQ, Precipitation of Driest Quarter; PWaQ, Precipitation of Warmest Quarter; PWQ, Precipitation of Coldest Quarter.

**Additional file 3.** Available values are plotted to demonstrate the key environmental parameters and the ecological niche occupied by VACV according to MaxENT predictions (black), 87 Brazilian outbreaks (red), and background (grey) of the remaining 15 bioclim layers not shown in Fig. 4. On the Y-axis is the number of pixels for each variable and the X-Axis, the bioclim variable indicated.

## Abbreviations

VACV: Vaccinia virus
ENM: ecological niche model
DPI: days post-inoculation
PC: principal components
