# Supplementary material for: Ecological niche modeling to determine potential niche of Vaccinia virus: a case only study
Source: Int J Health Geogr. 2017 Aug 7;16:28. doi: 10.1186/s12942-017-0100-1 (PMC5547515; doi:10.1186/s12942-017-0100-1)
Supplement: Supplementary file 1 — Additional file 1. List of Brazilian outbreaks used to generate ENM, with references. [file 12942_2017_100_MOESM1_ESM.docx]

| **State** | **Municipality** | **Year** | **Publication** |
| --- | --- | --- | --- |
| Bahia | Itiúba | 2014 | Abrahão 2016 [1] |
| Espírito Santo | Alegre | 2006 | Damaso 2007 [2] |
| Espírito Santo | Alegre | 2002-2005 * | Donatele 2007 [3] |
| Espírito Santo | Atílio Vivacqua | 2002-2005 * | Donatele 2007 [3] |
| Espírito Santo | Cachoeiro de Itapemirim | 2002-2005 * | Donatele 2007 [3] |
| Espírito Santo | Castelo | 2002-2005 * | Donatele 2007 [3] |
| Espírito Santo | Itapemirim | 2002-2005 * | Donatele 2007 [3] |
| Espírito Santo | Piúma | 2002-2005 * | Donatele 2007 [3] |
| Espírito Santo | Presidente Kennedy | 2002-2005 * | Donatele 2007 [3] |
| Espírito Santo | Rio Novo do Sul | 2002-2005 * | Donatele 2007 [3] |
| Goiás | Jataí | 2011 | de Sant'Ana 2013 [4] |
| Goiás | Morro Agudo | 2003 | Nagasse-Sugahara 2004 [5] |
| Goiás | Riánopolis | 2003 | Nagasse-Sugahara 2004 [5] |
| Maranhaõ | Açalăndia | 2009 | Oliveira 2013 [6] |
| Mato Grosso | Araputanga | 2007 | Promed Mail 2007 [7] |
| Mato Grosso | Figueirópolis d'oeste | 2007 | Promed Mail 2007 [7] |
| Minas Gerais | Antônio Prado | 2001 | Lobato 2005 [8] |
| Minas Gerais | Argirita | 2001 | Lobato 2005 [8] |
| Minas Gerais | Astolfo Dutra | 2001 | Lobato 2005 [8] |
| Minas Gerais | Bambuí | 2005 | de Trindade and Madureira 2007 [9] |
| Minas Gerais | Barão de Monte Alto | 2001 | Lobato 2005 [8] |
| Minas Gerais | Cantagalo | 1999, 2006 ** | Simonetti 2007 [10], Damaso 2000 [11], Silva-Fernandes 2009 [12] |
| Minas Gerais | Cataguases | 2001 | Lobato 2005 [8] |
| Minas Gerais | Delfim Moreira | 2001-2003 * | Nagasse-Sugahara [5] 2004 [5] |
| Minas Gerais | Dona Eusébia | 2001 | Lobato 2005 [8] |
| Minas Gerais | Eugenopolis | 2001 | Lobato 2005 [8] |
| Minas Gerais | Guarani | 2001 | de Souza Trindade 2007 [9], Lobato 2005 [8] |
| Minas Gerais | Juiz de Fora | 2001-2003 * | Nagasse-Sugahara 2004 [5] |
| Minas Gerais | Laranjal | 2001, 2000 ** | Lobato 2005 [8], Damaso 2007 [2] |
| Minas Gerais | Leopoldina | 2001 | Lobato 2005 [8] |
| Minas Gerais | Medeiros | 2005 | Personal comm. 2007 Trindade and Madureira^◆^ |
| Minas Gerais | Mercês | 2005 | Personal comm. 2007 Trindade and Madureira^◆^ |
| Minas Gerais | Miraí | 2001 | Lobato 2005 [8] |
| Minas Gerais | Muriaé | 2001 | Lobato 2005 [8], de Souza Trindade 2007 [9], |
| Minas Gerais | Palma | 2000 | Damaso 2007 [2] |
| Minas Gerais | Passa-Tempo | 2003 | Leite 2005 [13] |
| Minas Gerais | Piau | 2001 | Lobato 2005 [8] |
| Minas Gerais | Pirapetinga | 2001 | Lobato 2005 [8] |
| Minas Gerais | Piraúba | 2001 | Lobato 2005 [8] |
| Minas Gerais | Rio Pomba | 2001, 2005** | Lobato 2005 [8], Personal comm. 2007 Trindade and Madureira^◆^ |
| Minas Gerais | Santana de Cataguases | 2000, 2001 ** | Damaso 2007 [2], Lobato 2005 [8] |
| Minas Gerais | Serro | 2005 | Personal comm. 2007 Trindade and Madureira^◆^ |
| Minas Gerais | Ubá | 2001 | Lobato 2005 [8] |
| Minas Gerais | Vieiras | 2001 | Damaso 2007 [2], Lobato 2005 [8] |
| Minas Gerais | Volta Grande | 2001 | Lobato 2005 [8] |
| Rio de Janeiro | Aperibé | 1999 -2007 * | Simonetti 2007 [10] |
| Rio de Janeiro | Barra do Piraí | 2006 -2007 * | Costa 2007 [14] |
| Rio de Janeiro | Barra Mansa | 2001 | Damaso 2007 [2] |
| Rio de Janeiro | Cambuci | 1999-2007 * | Simonetti 2007 [10] |
| Rio de Janeiro | Campos de Goytacazes "Campos" | 2003, 2004 ** | Damaso 2007 [2], Silva-Fernandes, 2009 [12] |
| Rio de Janeiro | Cordeiro | 2006 | Simonetti 2007 [10], Silva-Fernandes, 2009 [12] |
| Rio de Janeiro | Italva | 2006 | Damaso 2007 [2] |
| Rio de Janeiro | Miracema | 1999, 2006 ** | Damaso 2007 [2], Simonetti 2007 [10], Silva-Fernandes, 2009 [12] |
| Rio de Janeiro | Natividade | 2006 | Silva-Fernandes, 2009 [12] |
| Rio de Janeiro | Piraí | 2006-2007 * | Costa 2007 [14] |
| Rio de Janeiro | Quatis | 2001 | Damaso 2007 [2] |
| Rio de Janeiro | Resende | 2006-2007 * | Costa 2007 [14] |
| Rio de Janeiro | Rio Claro | 2006-2007 * | Costa 2007 [14] |
| Rio de Janeiro | Rio das Flores | 2006-2007 * | Costa 2007 [14] |
| Rio de Janeiro | Santo Antônio de Pádua | 1999 | Simonetti 2007 [10], Damaso 2000 [11], Schatzmayr 2011 [15] |
| Rio de Janeiro | São Francisco de Itabapoana | 2002 | Silva-Fernandes 2009 [12] |
| Rio de Janeiro | São Sebastião do Alto | 2006 | Silva-Fernandes 2009 [12] |
| Rio de Janeiro | São Sebastião do Paraíba  (District) | 1999 | Damaso 2007 [2] |
| Rio de Janeiro | Valença (Valencia) | 2006-2007 * | Costa 2007 [14] |
| Rio de Janeiro | Varre-Sai | 2006 | Silva-Fernandes 2009 [12] |
| Rio Grande do Sul | Pelotas | 2008 | Campos 2011 [16] |
| São Paulo | Araçatuba | Unknown | de Souza Trindade 2007 [9] |
| São Paulo | Areias | 2001 -2003 * | Nagasse-Sugahara 2004 [5] |
| São Paulo | Cachoeira Paulista | 2001 -2003 * | Nagasse-Sugahara 2004 [5] |
| São Paulo | Canas | 2001-2003 * | Nagasse-Sugahara 2004 [5] |
| São Paulo | Cruziero | 2001-2003 * | Nagasse-Sugahara 2004 [5] |
| São Paulo | Guaratinguetá | 2001-2003 * | Nagasse-Sugahara 2004 [5] |
| São Paulo | Itatinga | 2009, 2012 ** | Megid 2012 [17], Peres 2016 [18] |
| São Paulo | Lagoinha | 2001-2003 * | Nagasse-Sugahara 2004 [5] |
| São Paulo | Lavrinhas | 2001-2003 * | Nagasse-Sugahara 2004 [5] |
| São Paulo | Lorena | 2001-2003 * | Nagasse-Sugahara 2004 [5] |
| São Paulo | Piquete | 2001-2003 * | Nagasse-Sugahara 2004 [5] |
| São Paulo | Silveiras | 2001-2003 * | Nagasse-Sugahara 2004 [5] |
| São Paulo | Torre de Pedra | 2007, 2010 ** | Megid 2012 [17], Peres 2013 [19] |

* Outbreak occurred at some point in time during this date range

**Outbreaks occurred during each year listed

^◆^ Outbreak was documented via personal communications between Giliane de Souza

Trindade and M.C. Madureira in 2007

[1] J. Santos Abrahao *et al.*, “Detection of Vaccinia virus during an outbreak of exanthemous oral lesions in Brazilian equids.,” *Equine Vet. J.*, Feb. 2016.

[2] C. R. A. Damaso, S. A. Reis, D. M. Jesus, P. S. F. Lima, and N. Moussatche, “A PCR-based assay for detection of emerging vaccinia-like viruses isolated in Brazil.,” *Diagn. Microbiol. Infect. Dis.*, vol. 57, no. 1, pp. 39–46, Jan. 2007.

[3] D. M. Donatele, C. E. P. F. Travassos, J. de Almeida Leite, and E. G. Kroon, “Epidemiologia da poxvirose bovina no Estado do Espírito Santo, Brasil,” *Braz. J. Vet. Res. Anim. Sci.*, vol. 44, no. 4, pp. 275–282, 2007.

[4] F. J. F. de Sant’Ana *et al.*, “Coinfection by Vaccinia virus and an Orf virus-like parapoxvirus in an outbreak of vesicular disease in dairy cows in midwestern Brazil.,” *J. Vet. Diagn. Investig. Off. Publ. Am. Assoc. Vet. Lab. Diagn. Inc*, vol. 25, no. 2, pp. 267–272, Mar. 2013.

[5] T. K. Nagasse-Sugahara *et al.*, “Human vaccinia-like virus outbreaks in Sao Paulo and Goias States, Brazil: virus detection, isolation and identification.,” *Rev. Inst. Med. Trop. Sao Paulo*, vol. 46, no. 6, pp. 315–322, Dec. 2004.

[6] D. B. Oliveira *et al.*, “Group 1 Vaccinia virus zoonotic outbreak in Maranhao State, Brazil.,” *Am. J. Trop. Med. Hyg.*, vol. 89, no. 6, pp. 1142–1145, Dec. 2013.

[7] ProMED-mail., “VACCINIA, BOVINE, HUMAN - BRAZIL (MATO GROSSO),” ProMED-mail post, Nov. 2007.

[8] Z. I. Lobato *et al.*, “Surto de varíola bovina causada pelo vírus Vaccinia na região da Zona da Mata Mineira,” *Arq Bras Med Vet Zootec*, vol. 57, no. 4, pp. 423–429, 2005.

[9] G. de Souza Trindade *et al.*, “Zoonotic vaccinia virus infection in Brazil: clinical description and implications for health professionals.,” *J. Clin. Microbiol.*, vol. 45, no. 4, pp. 1370–1372, Apr. 2007.

[10] B. R. Simonetti *et al.*, “ANIMAL INFECTIONS BY VACCINIA-LIKE VIRUSES IN THE STATE OF RIO DE JANEIRO: 1-NORTHWESTERN REGION.,” 2007.

[11] C. R. Damaso, J. J. Esposito, R. C. Condit, and N. Moussatche, “An emergent poxvirus from humans and cattle in Rio de Janeiro State: Cantagalo virus may derive from Brazilian smallpox vaccine.,” *Virology*, vol. 277, no. 2, pp. 439–449, Nov. 2000.

[12] A. T. Silva-Fernandes *et al.*, “Natural human infections with Vaccinia virus during bovine vaccinia outbreaks.,” *J. Clin. Virol. Off. Publ. Pan Am. Soc. Clin. Virol.*, vol. 44, no. 4, pp. 308–313, Apr. 2009.

[13] J. A. Leite *et al.*, “Passatempo virus, a vaccinia virus strain, Brazil.,” *Emerg. Infect. Dis.*, vol. 11, no. 12, pp. 1935–1938, Dec. 2005.

[14] R. V. C. Costa *et al.*, “Animal infections by vaccinia-like viruses in the state of Rio de Janeiro: 2-Paraíba river valley,” *Virus Rev Res*, vol. 12, pp. 37–42, 2007.

[15] H. G. Schatzmayr, R. V. C. Costa, M. C. R. Goncalves, P. S. D’Andrea, and O. M. Barth, “Human and animal infections by vaccinia-like viruses in the state of Rio de Janeiro: a novel expanding zoonosis.,” *Vaccine*, vol. 29 Suppl 4, pp. D65–69, Dec. 2011.

[16] R. K. Campos *et al.*, “Assessing the variability of Brazilian Vaccinia virus isolates from a horse exanthematic lesion: coinfection with distinct viruses.,” *Arch. Virol.*, vol. 156, no. 2, pp. 275–283, Feb. 2011.

[17] J. Megid *et al.*, “Vaccinia virus zoonotic infection, Sao Paulo State, Brazil.,” *Emerg. Infect. Dis.*, vol. 18, no. 1, pp. 189–191, Jan. 2012.

[18] M. G. Peres *et al.*, “Dogs and Opossums Positive for Vaccinia Virus during Outbreak Affecting Cattle and Humans, Sao Paulo State, Brazil.,” *Emerg. Infect. Dis.*, vol. 22, no. 2, pp. 271–273, Feb. 2016.

[19] M. G. Peres *et al.*, “Serological study of vaccinia virus reservoirs in areas with and without official reports of outbreaks in cattle and humans in Sao Paulo, Brazil.,” *Arch. Virol.*, vol. 158, no. 12, pp. 2433–2441, Dec. 2013.
