# Supplementary material for: Ecological niche modeling to determine potential niche of Vaccinia virus: a case only study
Source: Int J Health Geogr. 2017 Aug 7;16:28. doi: 10.1186/s12942-017-0100-1 (PMC5547515; doi:10.1186/s12942-017-0100-1)
Supplement: Supplementary file 2 — Additional file 2. Summary statistics of key bioclim values as predicted by MaxENT modeling, using a 10% threshold, and what is indicated at the points of VCAV occurrence in Brazil and Colombia. Temperatures are reported in degrees Fahrenheit and precipitation in mm. AMT, annual mean temperature; MDR, mean diurnal range; ISO, isothermability (Bio2/Bio7)*(100); TS, Temperature Seasonality (standard deviation *100); MTWaM; maximum temperature of the warmest month; MTCM, minimum temperature of the coldest month; TAR, Temperature Annual Range (Bio5-Bio6); MTWQ, Mean Temperature of Wettest Quarter; MTDQ, Mean Temperature of Driest Quarter; MTWaQ, Mean Temperature of Warmest Quarter; MTCQ, Mean Temperature of Coldest Quarter; AP, annual precipitation; PWM, precipitation of the wettest month; PDM, precipitation of the driest month; PS, Precipitation Seasonality (Coefficient of Variation); PWQ, Precipitation of Wettest Quarter; PDQ, Precipitation of Driest Quarter; PWaQ, Precipitation of Warmest Quarter; PWQ, Precipitation of Coldest Quarter. [file 12942_2017_100_MOESM2_ESM.docx]

| **Locality** |  | **AMT** | **MDR** | **ISO** | **TS** | **MTWaM** | **MTCM** | **TAR** | **MTWQ** | **MTDQ** | **MTWaQ** | **MTCQ** | **AP** | **PWM** | **PDM** | **PS** | **PWQ** | **PDQ** | **PWaQ** | **PCQ** |
| --- | --- | --- | --- | --- | --- | --- | --- | --- | --- | --- | --- | --- | --- | --- | --- | --- | --- | --- | --- | --- |
| **MaxENT** | **Min** | 12.50 | 6.60 | 4.20 | 38.00 | 20.20 | 1.50 | 9.60 | 12.60 | 9.90 | 14.60 | 9.50 | 468.00 | 68.00 | 0.00 | 7.00 | 199.00 | 0.00 | 32.00 | 1.00 |
|  | **Max** | 28.30 | 15.70 | 8.30 | 377.00 | 36.30 | 23.10 | 23.50 | 28.70 | 29.80 | 29.80 | 27.10 | 3571.00 | 585.00 | 162.00 | 116.00 | 1546.00 | 558.00 | 1022.00 | 1379.00 |
|  | **Mean** | 22.53 | 11.74 | 6.54 | 185.27 | 30.90 | 12.99 | 17.91 | 23.72 | 20.69 | 24.50 | 19.90 | 1494.45 | 248.12 | 30.30 | 61.56 | 671.68 | 113.49 | 431.30 | 200.23 |
|  | **SD** | 2.87 | 1.46 | 0.59 | 72.77 | 2.56 | 3.78 | 2.27 | 2.61 | 3.68 | 2.24 | 3.62 | 415.09 | 70.64 | 32.71 | 23.42 | 193.42 | 110.20 | 164.88 | 236.59 |
| **Brazil** | **Min** | 15.50 | 8.90 | 4.80 | 33.50 | 23.30 | 4.20 | 14.40 | 15.40 | 12.30 | 18.00 | 12.30 | 668.00 | 97.00 | 4.00 | 17.00 | 255.00 | 21.00 | 138.00 | 44.00 |
|  | **Max** | 25.90 | 13.60 | 7.80 | 365.60 | 33.30 | 18.70 | 21.00 | 26.10 | 26.00 | 26.50 | 25.50 | 1814.00 | 356.00 | 79.00 | 86.00 | 932.00 | 283.00 | 850.00 | 396.00 |
|  | **Mean** | 21.47 | 11.64 | 6.29 | 207.03 | 29.96 | 11.57 | 18.40 | 23.33 | 18.83 | 23.88 | 18.61 | 1340.60 | 246.91 | 20.69 | 68.96 | 658.60 | 75.21 | 528.54 | 90.23 |
|  | **SD** | 1.93 | 1.01 | 0.37 | 40.41 | 1.96 | 2.69 | 1.60 | 1.80 | 2.34 | 1.73 | 2.28 | 182.11 | 44.90 | 9.84 | 11.28 | 116.85 | 33.94 | 142.93 | 43.25 |
| **Colombia** | **Min** | 15.40 | 9.00 | 7.50 | 35.20 | 21.40 | 8.90 | 11.00 | 15.60 | 14.80 | 15.90 | 14.50 | 1089.00 | 196.00 | 11.00 | 27.00 | 461.00 | 77.00 | 99.00 | 111.00 |
|  | **Max** | 26.80 | 9.90 | 8.70 | 91.20 | 34.10 | 21.30 | 12.80 | 25.70 | 27.50 | 28.00 | 25.60 | 5081.00 | 753.00 | 157.00 | 63.00 | 2081.00 | 569.00 | 862.00 | 2002.00 |
|  | **Mean** | 23.22 | 9.64 | 8.10 | 58.24 | 29.54 | 17.68 | 11.86 | 22.78 | 23.48 | 23.90 | 22.40 | 2817.20 | 406.80 | 71.60 | 49.00 | 1096.20 | 299.80 | 459.00 | 973.60 |
|  | **SD** | 4.68 | 0.38 | 0.47 | 20.37 | 4.95 | 5.19 | 0.77 | 4.29 | 5.17 | 4.79 | 4.68 | 1575.05 | 210.65 | 58.66 | 14.14 | 612.82 | 206.77 | 294.33 | 681.11 |
