# Supplementary material for: Ecological niche modeling to determine potential niche of Vaccinia virus: a case only study
Source: Int J Health Geogr. 2017 Aug 7;16:28. doi: 10.1186/s12942-017-0100-1 (PMC5547515; doi:10.1186/s12942-017-0100-1)
Supplement: Supplementary file 3 [file 12942_2017_100_MOESM3_ESM.docx]

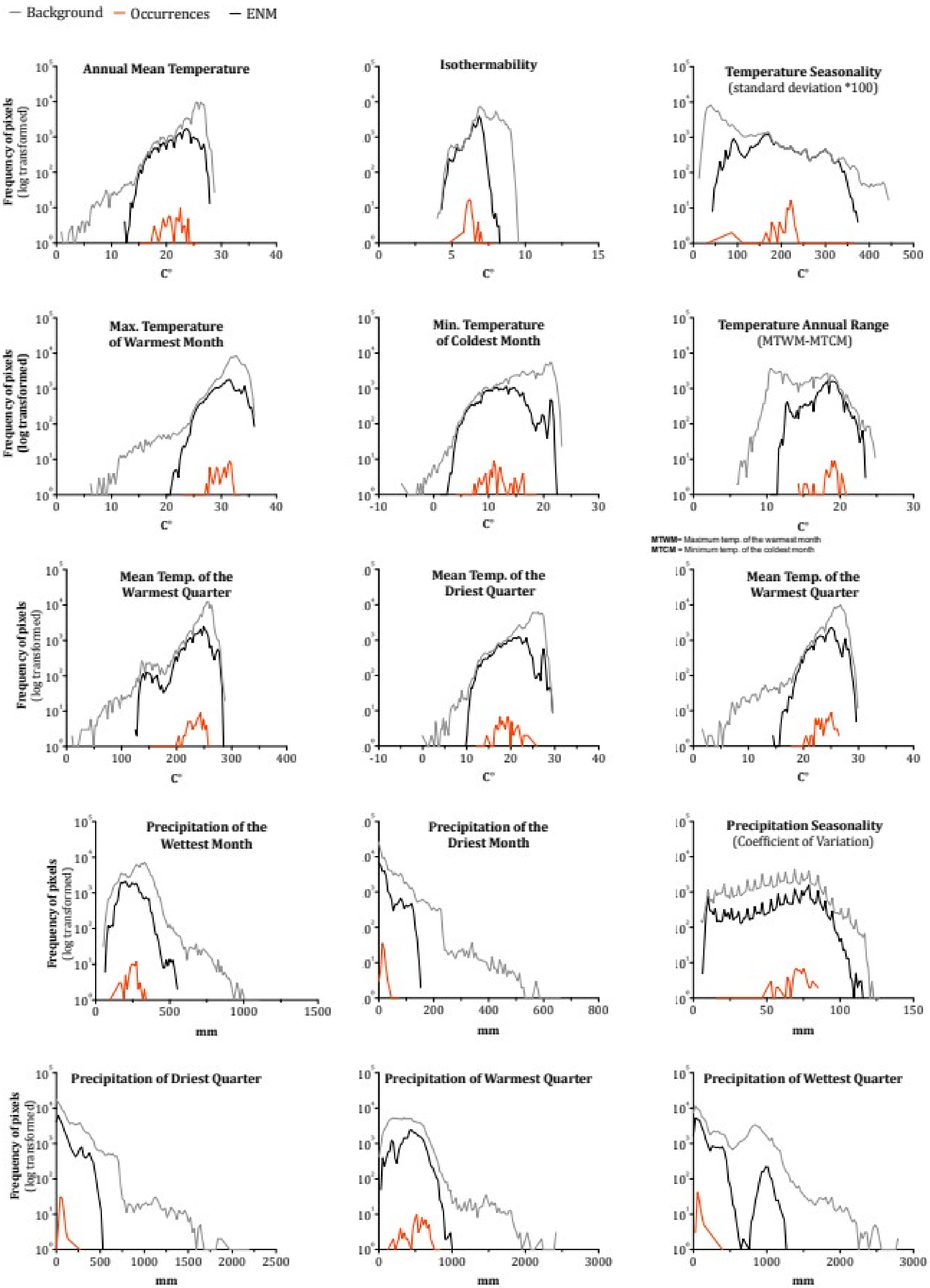

Background
Occurrences
ENM
Frequency of pixels (log transformed)
Annual Mean Temperature
Isothermability
Temperature Seasonality (standard deviation *100)
Max. Temperature of Warmest Month
Min. Temperature of Coldest Month
Temperature Annual Range (MTWM-MTCM)
MTWM= Maximum temp. of the warmest month
MTCM = Minimum temp. of the coldest month
Mean Temp. of the Warmest Quarter
Mean Temp. of the Driest Quarter
Mean Temp. of the Warmest Quarter
Precipitation of the Wettest Month
Precipitation of the Driest Month
Precipitation Seasonality (Coefficient of Variation)
Precipitation of Driest Quarter
Precipitation of Warmest Quarter
Precipitation of Wettest Quarter
C°
mm
